# Baseline Prediabetes, Hyperglycemia, and Incident Dental Outcomes Among Peruvian Formal-Sector Workers: A Retrospective Occupational Cohort Study

**DOI:** 10.3390/dj14090536

**Published:** 2026-08-27

**Authors:** Víctor Juan Vera-Ponce, Jhosmer Ballena-Caicedo, Nelly Del Carmen Villegas Ampuero, Julio David Sagastegui Jauregui, Fiorella E. Zuzunaga-Montoya

**Affiliations:** 1 Facultad de Medicina (FAMED), Universidad Nacional Toribio Rodríguez de Mendoza de Amazonas (UNTRM), Chachapoyas 01001, Peru; 7330178022@untrm.edu.pe (J.B.-C.); fiorella.zuzunaga@untrm.edu.pe (F.E.Z.-M.); 2 Escuela de Odontología, Universidad Nacional Toribio Rodríguez de Mendoza de Amazonas (UNTRM), Chachapoyas 01001, Peru; nelly.villegas@untrm.edu.pe (N.D.C.V.A.); julio.sagastegui.epg@untrm.edu.pe (J.D.S.J.)

**Keywords:** prediabetes, hyperglycemia, dental caries, gingivitis, occupational health, cohort studies, Peru

## Abstract

**Background/Objectives:** Early glycemic abnormalities may identify adults at increased oral health risk, but longitudinal evidence from Latin American occupational populations is limited. We evaluated whether screening-based baseline prediabetes and hyperglycemia were associated with recorded incident dental outcomes among Peruvian formal-sector workers. **Methods:** We conducted a retrospective cohort study using occupational health records from 2013–2021. The primary outcome was a composite incident dental outcome, defined as incident caries or gingivitis; secondary outcomes were incident caries and incident gingivitis separately. Screening-based baseline prediabetes was compared with normoglycemia after excluding diabetes, whereas screening-based baseline hyperglycemia included prediabetes or diabetes and was compared with normoglycemia. Cox proportional hazards models were adjusted for age, sex, occupation type, smoking, and alcohol consumption, with inverse-probability weighted analyses for exposure and follow-up. **Results:** Among 79,660 workers, 12,049 (15.1%) had longitudinal dental follow-up. After outcome-specific exclusions and glycemic classification, the hyperglycemia analysis included 10,050 workers and 345 composite dental events, and the prediabetes analysis included 9804 workers and 335 events. Prediabetes was associated with higher hazards of the composite outcome (adjusted HR 1.61; 95% CI 1.20–2.17) and incident caries (adjusted HR 1.94; 95% CI 1.42–2.65). Hyperglycemia showed similar associations with the composite outcome (adjusted HR 1.55; 95% CI 1.17–2.05) and incident caries (adjusted HR 1.87; 95% CI 1.39–2.52). No increased hazard of incident gingivitis was observed. Estimates were directionally stable after obesity adjustment and inverse-probability weighting. **Conclusions:** Screening-based baseline prediabetes and hyperglycemia were associated with higher recorded incidence of dental outcomes, mainly incident caries, among workers with longitudinal dental follow-up. Limited dental follow-up reduces generalizability and may reflect heterogeneous implementation of dental surveillance. Because proportional hazards diagnostics suggested time-varying associations for the composite outcome and caries, Cox estimates should be interpreted as summary associations over follow-up.

## 1. Introduction

Oral diseases represent a persistent and largely preventable global health burden. The World Health Organization estimates that they affect nearly 3.7 billion people and recognizes untreated caries in permanent teeth as the most frequent health condition according to the Global Burden of Disease 2021. This burden is not limited to local damage: caries, periodontal diseases, tooth loss, and their functional consequences are associated with pain, difficulty eating, absenteeism, and direct and indirect costs for health systems and productivity. Recent global analyses confirm that the burden of oral conditions has remained high over recent decades, with substantial regional variation and without reductions proportional to progress achieved in other areas of public health [1,2].

The relationship between oral health and noncommunicable diseases is relevant because both groups of conditions share behavioral, metabolic, and social determinants. Free-sugar intake is a common risk factor for dental caries, obesity, and metabolic disorders, whereas smoking, alcohol consumption, oral hygiene, access to dental care, and socioeconomic position shape the population distribution of oral outcomes. In addition, diabetes and periodontal disease have been described as reciprocally linked conditions, although the magnitude and consistency of associations vary by oral outcome, type of diabetes, glycemic control, and study design. In this context, the available longitudinal evidence is more developed for periodontitis than for caries, and is even more limited for intermediate states such as prediabetes or screening-detected hyperglycemia [1,3,4].

In Latin America and the Caribbean, this intersection is important because of the epidemiologic transition, the expansion of cardiometabolic risk factors, and persistent inequalities in access to preventive services. In the Region of the Americas, the age-standardized prevalence of diabetes among adults was estimated at 13.1% in 2022, equivalent to 112 million people, representing an 85% increase relative to 1990. In Peru, regional health profiles have documented a high burden of overweight and obesity and an increase in the reported prevalence of diabetes mellitus among adults between 2000 and 2014. A recent systematic review and meta-analysis estimated pooled prevalences of diabetes and prediabetes in Peru of 7.47% and 10.66%, respectively, underscoring the need to study not only established diabetes but also early glycemic abnormalities [5,6,7].

The Peruvian context also shows a substantial burden of oral disease. The World Health Organization oral health country profile estimated that Peru had a prevalence of untreated caries in permanent teeth of 38.2% among persons aged 5 years or older, severe periodontal disease of 19.2% among persons aged 15 years or older, and edentulism of 14.6% among adults aged 20 years or older. The same profile reported structural limitations of the system, including a dentist density of 1.5 per 10,000 population and costs attributable to oral diseases. Population-based Peruvian studies have also described socioeconomic inequalities in the use of oral health services, reinforcing the need for local evidence linking metabolic factors with longitudinally observed oral outcomes [8,9].

Most available evidence on glycemic abnormalities and oral disease focuses on established diabetes, periodontal disease, or cross-sectional designs, limiting temporal inference on early glycemic states and incident dental outcomes in working-age adults. Occupational health records are informative because they may contain repeated metabolic and dental evaluations in adults who are often underrepresented in preventive oral health programs. However, the availability of longitudinal dental follow-up may vary across employers and occupational protocols. In this database, only workers with longitudinal dental records could contribute to incident dental outcome analyses. The absence of such records indicates limited availability of longitudinal dental outcome data within the occupational record system, and these workers cannot be assumed to represent a directly comparable unsupervised counterfactual group. Therefore, this study aimed to estimate the association between screening-based baseline prediabetes and hyperglycemia and incident dental outcomes among Peruvian formal-sector workers with longitudinal dental follow-up, and to describe the extent of dental follow-up availability in the occupational records.

## 2. Materials and Methods

### 2.1. Study Design

We conducted an observational longitudinal retrospective cohort study using secondary records from occupational health evaluations of formal-sector workers in Peru. The observation period corresponded to evaluations recorded between 2013 and 2021, with follow-up available until the last occupational evaluation registered in the database. The analytical objective was to estimate the association between baseline screening-based glycemic abnormality and incident dental outcomes during follow-up. The manuscript was prepared according to the STROBE recommendations for observational cohort studies [10].

### 2.2. Data Source and Study Population

The data source was an institutional database of occupational medical evaluations. Records included sociodemographic and occupational information, behavioral history, anthropometric measurements, blood pressure, dental evaluation, and biochemical tests when requested as part of the occupational evaluation. The unit of analysis was the worker evaluated at the baseline visit and eligible to be observed at a subsequent evaluation.

For the main analysis, eligible workers were those with positive follow-up time, baseline dental information, records of incident dental outcomes, and available baseline glycemic classification. Because the primary outcome was incident dental outcomes, workers with caries or gingivitis recorded at baseline were excluded from the composite outcome analysis. In secondary analyses by specific outcome, workers with prevalent caries at baseline were excluded from the incident caries analysis, and those with prevalent gingivitis at baseline were excluded from the incident gingivitis analysis. The availability of other laboratory tests, such as cholesterol or triglycerides, was not used as an inclusion criterion because these measurements depended on the administrative requests of each company and were not essential to the primary research question.

### 2.3. Exposures and Outcomes

The main exposures were screening-based baseline prediabetes and screening-based baseline hyperglycemia. Baseline prediabetes was analyzed against baseline normoglycemia, excluding baseline diabetes from that comparison. Baseline hyperglycemia was analyzed as a broader category that included baseline prediabetes or diabetes and was compared with normoglycemia. Both exposures were evaluated in separate models because hyperglycemia includes prediabetes and diabetes and therefore is not independent of prediabetes.

Operational definitions were based on screening fasting plasma glucose recorded at baseline in the occupational database. When these variables corresponded to baseline glucose, interpretation followed the usual fasting plasma glucose cutoffs: normoglycemia below 100 mg/dL, prediabetes between 100 and 125 mg/dL, and diabetes at 126 mg/dL or higher [11]. These categories should be interpreted as baseline screening-based glycemic categories rather than confirmed clinical diagnoses. Because a diagnosis of diabetes mellitus requires repeated testing on a different occasion, baseline diabetes was not considered a study outcome, and misclassification near diagnostic thresholds remains possible.

The primary outcome was a composite incident dental outcome, defined as the occurrence of incident caries or incident gingivitis during follow-up among workers free of both conditions at baseline. This composite was used as an operational measure of incident oral morbidity captured in occupational records and should not be interpreted as implying identical biological mechanisms for caries and gingivitis. Therefore, secondary outcome-specific analyses for incident caries and incident gingivitis were prespecified and interpreted as clinically more specific estimates. Outcomes were analyzed using the incidence variables recorded in the occupational database.

Follow-up time was expressed in years. For workers with an event, time at risk corresponded to the recorded follow-up time up to the evaluation at which the event was classified. For workers without an event, follow-up was censored at the last available evaluation. The database did not contain the exact clinical onset date of the dental event; therefore, estimates should be interpreted as associations with dental outcomes detected during the occupational follow-up interval, not as the exact biological onset date.

**Figure 1:**
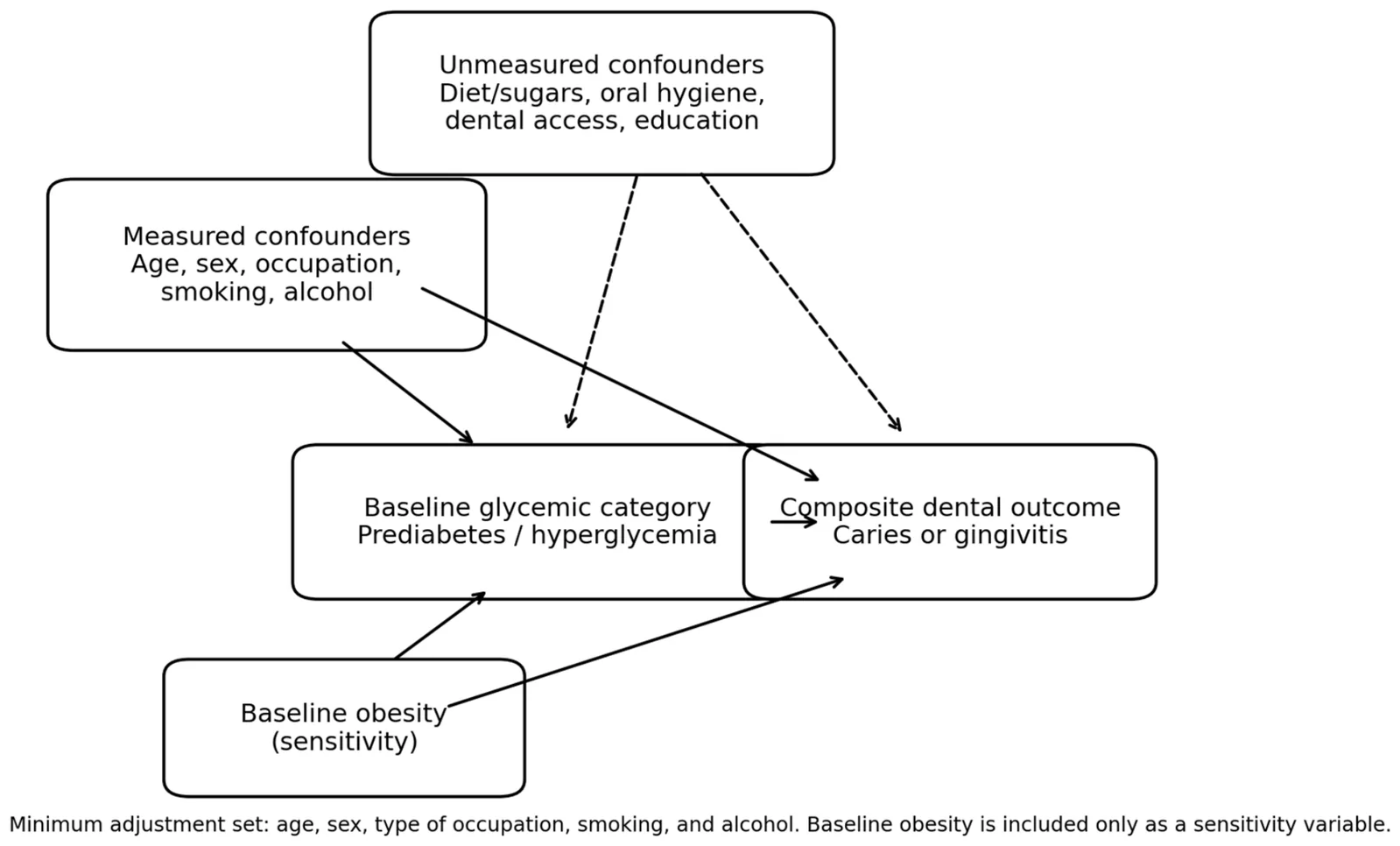
Proposed directed acyclic graph for screening-based baseline prediabetes/hyperglycemia and the composite dental outcome.

The adjustment set was defined before modeling by using a directed acyclic graph, based on epidemiologic assumptions regarding common causes of glycemic abnormality and incident dental outcomes. Directed acyclic graphs make causal assumptions explicit and help identify variables that should be controlled to reduce confounding under those assumptions [12]. The main model included baseline age, sex, occupation type, baseline smoking, and baseline alcohol consumption. These covariates were selected because of their plausibility as common causes of glycemic exposure and dental outcomes and because they were available at the baseline evaluation (Figure 1).

We did not adjust for variables measured at the end of follow-up or for variables that could have been affected by baseline exposure. Baseline obesity was treated as a methodologically debatable variable: it may act as a common cause of glycemic abnormality and dental outcomes, but it is also part of the metabolic constellation that the study aimed to describe. Therefore, the main analysis did not include it as a mandatory covariate, and a sensitivity analysis with additional adjustment for baseline obesity was specified to assess the stability of the estimates.

### 2.4. Variable Measurement Procedures

Occupational evaluations were performed as part of periodic medical care for formal-sector workers. At the baseline evaluation, sociodemographic data, employment information, medical history, and health behaviors were recorded using a structured occupational medical history. Dental variables were obtained during a clinical evaluation performed by a certified dental professional. The presence of dental caries and gingivitis was determined from the dental examination recorded in the occupational medical record, not by self-report. Prevalent composite dental outcome was defined as the presence of caries or gingivitis at the baseline evaluation, whereas incident composite dental outcome was defined as the record of caries or gingivitis during follow-up among workers free of both conditions at baseline.

Dental outcomes were obtained from routine occupational dental examinations rather than standardized research examinations. The analytic database available for this study did not include examiner identifiers, formal inter- or intra-examiner calibration results, radiographic findings, periodontal probing measurements, dental indices such as DMFT or ICDAS, or gingival index scores. Therefore, caries and gingivitis should be interpreted as clinician-recorded outcomes detected during occupational evaluations. This may introduce outcome misclassification, particularly for early caries lesions and mild gingival inflammation.

Anthropometric measurements included weight and height, obtained with the worker wearing light clothing and no shoes, and body mass index was calculated as weight in kilograms divided by height in meters squared. Baseline glucose was measured in a venous sample obtained by venipuncture after a minimum fasting period of 8 h and a maximum of 12 h. Participants were classified according to baseline glycemic status using fasting glucose, including normoglycemia, prediabetes, and hyperglycemia. Smoking and alcohol consumption were recorded through a structured interview as consumption in the previous 30 days. These behavioral variables were self-reported; however, workers signed the corresponding occupational documentation declaring the truthfulness of the information provided. Occupation type was recorded according to declared and documented employment information during the occupational evaluation and was subsequently categorized for analysis.

### 2.5. Statistical Analysis

Baseline characteristics were summarized using means and standard deviations for approximately symmetrically distributed continuous variables, medians and interquartile ranges for skewed variables, and absolute frequencies and percentages for categorical variables. Crude incidence rates were calculated by dividing the number of events by person-years of follow-up and were expressed per 1000 person-years. The 95% confidence intervals for rates were calculated using the Poisson distribution for events observed in person-time.

To characterize potential selection into the analytic cohort, baseline characteristics were compared between workers with and without longitudinal dental follow-up in the reduced database. Among workers with longitudinal dental follow-up and free of the baseline composite dental outcome, characteristics were also compared between those with and without baseline glycemic classification, because this exclusion defined entry into the glycemic analytic cohort. Differences were summarized using absolute standardized mean differences rather than formal hypothesis testing because of the large sample size.

The primary analysis used Cox proportional hazards models to estimate hazard ratios [13] and 95% confidence intervals. This choice was justified because the research question was longitudinal, outcomes were incident, and individual follow-up time was available. The time variable was follow-up recorded in years. Models were adjusted for the set defined by the directed acyclic graph. Prediabetes and hyperglycemia were modeled separately, and each outcome was analyzed in an outcome-specific population at risk. Hazard ratios should be interpreted as associations between baseline glycemic abnormality and incident detection of dental outcomes during occupational follow-up.

The proportional hazards assumption was evaluated using Schoenfeld residual-based tests for the exposure term and for the global model, complemented by inspection of time-dependent patterns in residuals. Because residual-based diagnostics suggested possible non-proportionality in models for the composite dental outcome and incident caries, exploratory time-stratified Cox models were fitted using a 2-year cut point, approximately corresponding to the median follow-up, estimating separate hazard ratios for the first two years and for follow-up beyond two years.

As a complementary analysis, inverse-probability weighted models were estimated. Exposure weights were estimated using baseline age, sex, occupation type, smoking in the past 30 days, and alcohol consumption in the past 30 days. Follow-up weights were estimated using the same baseline covariates and the corresponding baseline glycemic exposure indicator. Stabilized combined IPW/IPCW weights were truncated at the 1st and 99th percentiles to reduce the influence of extreme weights. Positivity was assessed descriptively through propensity score and weight distributions, and covariate balance after weighting was evaluated using standardized mean differences. The combination of exposure and follow-up weights was used to obtain marginal estimates under the assumptions of consistency, conditional exchangeability, positivity, and correct specification of the weighting models [14,15]. This analysis was considered a sensitivity analysis because the exposure was baseline and not time-dependent, and because the database did not include sufficiently complete repeated measurements of glycemic exposure or intermediate dental factors.

Additional sensitivity analyses were performed with adjustment for baseline obesity and with outcome-specific dental endpoints. Missing data were not imputed. Models used complete-case analysis for the indispensable variables in each comparison, explicitly preserving the distinction between absence of a nonrequired laboratory test and absence of a variable required to define the exposure. All analyses were two-sided, and *p* values below 0.05 were considered statistically significant. Analyses were documented using reproducible code in Stata version 17.0 and Python version 3.12.

### 2.6. Ethical Considerations

The study was based on secondary analysis of anonymized records derived from routine occupational medical evaluations. The database contained no direct personal identifiers, and workers were not contacted. Ethical review was waived by the Research Ethics Committee of the Universidad Nacional Toribio Rodríguez de Mendoza de Amazonas because the analysis used preexisting anonymized data, with no direct personal identifiers and no contact with workers (approval number: CIEI-N°0068; approval date: 20 June 2024).

## 3. Results

### 3.1. Sample Selection

The operational definitions of baseline prediabetes, baseline hyperglycemia, the composite dental outcome, incident caries, incident gingivitis, follow-up time, and covariates are summarized in Table S2. The reduced database available included 79,660 workers. Of these, only 12,049 (15.1%) had positive longitudinal dental follow-up and could therefore contribute to the incidence analysis; the remaining 67,611 workers (84.9%) had no available dental follow-up time or incident dental outcome records. This indicates limited availability of longitudinal dental outcome data within the occupational record system and may reflect heterogeneous implementation or recording of dental surveillance across employers. Because workers without longitudinal dental records could not be compared on incident dental outcomes, this issue represents both an operational finding regarding limited dental outcome availability and a potential source of selection bias that limits internal validity and generalizability. Among workers with follow-up, 198 had caries or gingivitis recorded at baseline and were excluded from the composite dental outcome analysis. The population at risk of the composite dental outcome comprised 11,851 workers. After excluding 1801 workers without available baseline glycemic classification, the analysis of hyperglycemia versus normoglycemia included 10,050 workers. For the analysis of prediabetes versus normoglycemia, 246 workers with baseline diabetes were additionally excluded; therefore, the analytic sample consisted of 9804 workers. The selection flow is shown in Figure S1 and detailed in tabular form in Table S3.

In the full database, there were no missing data for age, sex, smoking, alcohol consumption, or baseline composite dental outcome. Follow-up time and incident dental outcomes were available only among workers with longitudinal follow-up, corresponding to 12,049 non-missing observations. Baseline glucose and baseline hyperglycemia had 14,370 missing values, equivalent to 18.0% of the full database, whereas baseline prediabetes had 15,551 missing values, equivalent to 19.5%. Missingness for key variables is presented in Table S4. The distribution of follow-up time is shown in Figure S2.

Supplementary comparisons according to longitudinal dental follow-up availability and baseline glycemic classification availability are presented in Table S14. Workers with longitudinal dental follow-up differed from those without follow-up, particularly in age (37.2 vs. 32.9 years; absolute SMD = 0.390), prolonged sitting or seated work (48.5% vs. 38.6%; absolute SMD = 0.202), systolic blood pressure (111.3 vs. 108.7 mmHg; absolute SMD = 0.222), baseline hypertension (5.8% vs. 0.8%; absolute SMD = 0.279), and lipid-related measurements. Among workers with follow-up and no baseline composite dental outcome, availability of baseline glycemic classification differed most strongly by occupational composition, especially social services work (2.8% vs. 25.6%; absolute SMD = 0.693) and office work (38.7% vs. 22.2%; absolute SMD = 0.365), as well as waist circumference availability (84.1% vs. 94.4%; absolute SMD = 0.340), cholesterol availability (51.0% vs. 39.1%; absolute SMD = 0.241), and triglyceride availability (51.1% vs. 38.9%; absolute SMD = 0.248). These comparisons characterize potential selection into the analytic sample and analytic missingness, but they do not identify or quantify selection bias.

### 3.2. Baseline Characteristics

In the analytic population with baseline glycemic classification, 8315 workers were classified as normoglycemic, 1489 as prediabetic, and 246 as diabetic within the hyperglycemia group. Mean age was 37.4 years, with a standard deviation of 11.7 years. Mean age was 36.2 years in normoglycemia, 42.1 years in prediabetes, and 49.8 years in diabetes within hyperglycemia. Median follow-up was 2.1 years in the overall population and remained 2.1 years across the three glycemic groups. Baseline characteristics are presented in Table 1.

Male sex represented 85.5% of the total population, with proportions of 84.1% in normoglycemia, 92.3% in prediabetes, and 90.7% in diabetes within hyperglycemia. Physical/manual work was the most frequent occupational category, accounting for 55.4% of the total population, followed by office work at 38.7%. Current smoking in the previous 30 days was observed in 40.7% of the total population and in 39.5%, 45.5%, and 49.6% of the normoglycemia, prediabetes, and diabetes-within-hyperglycemia groups, respectively. Alcohol use in the previous 30 days was 49.8% in the total population.

Baseline body mass index-defined obesity was present in 22.3% of workers. Its frequency was 19.8% in normoglycemia, 33.2% in prediabetes, and 39.8% in diabetes within hyperglycemia. Baseline hypertension was observed in 6.1% of the total population, with frequencies of 5.0%, 9.9%, and 19.9% in normoglycemia, prediabetes, and diabetes within hyperglycemia, respectively. The composite dental outcome was observed in 345 workers, corresponding to 3.4% of the population with glycemic classification. Incident caries was observed in 280 workers and incident gingivitis in 107 workers.

### 3.3. Crude Incidence Rates and Adjusted Hazard Ratios

The crude incidence rate of the composite dental outcome was 12.22 per 1000 person-years in normoglycemia and 16.24 per 1000 person-years in prediabetes. In the hyperglycemia analysis, the incidence rate was 12.22 per 1000 person-years in normoglycemia and 16.15 per 1000 person-years in hyperglycemia. For incident caries, the incidence rate was 9.77 per 1000 person-years in normoglycemia and 15.00 per 1000 person-years in prediabetes. In the hyperglycemia comparison, the incident caries rate was 14.85 per 1000 person-years in the hyperglycemia group. Crude incidence rates by exposure and outcome are presented in Table 2.

For incident gingivitis, the crude incidence rate was 4.35 per 1000 person-years in normoglycemia and 2.81 per 1000 person-years in prediabetes. In the hyperglycemia comparison, the incidence rate was 2.62 per 1000 person-years in the hyperglycemia group.

**Figure 2:**
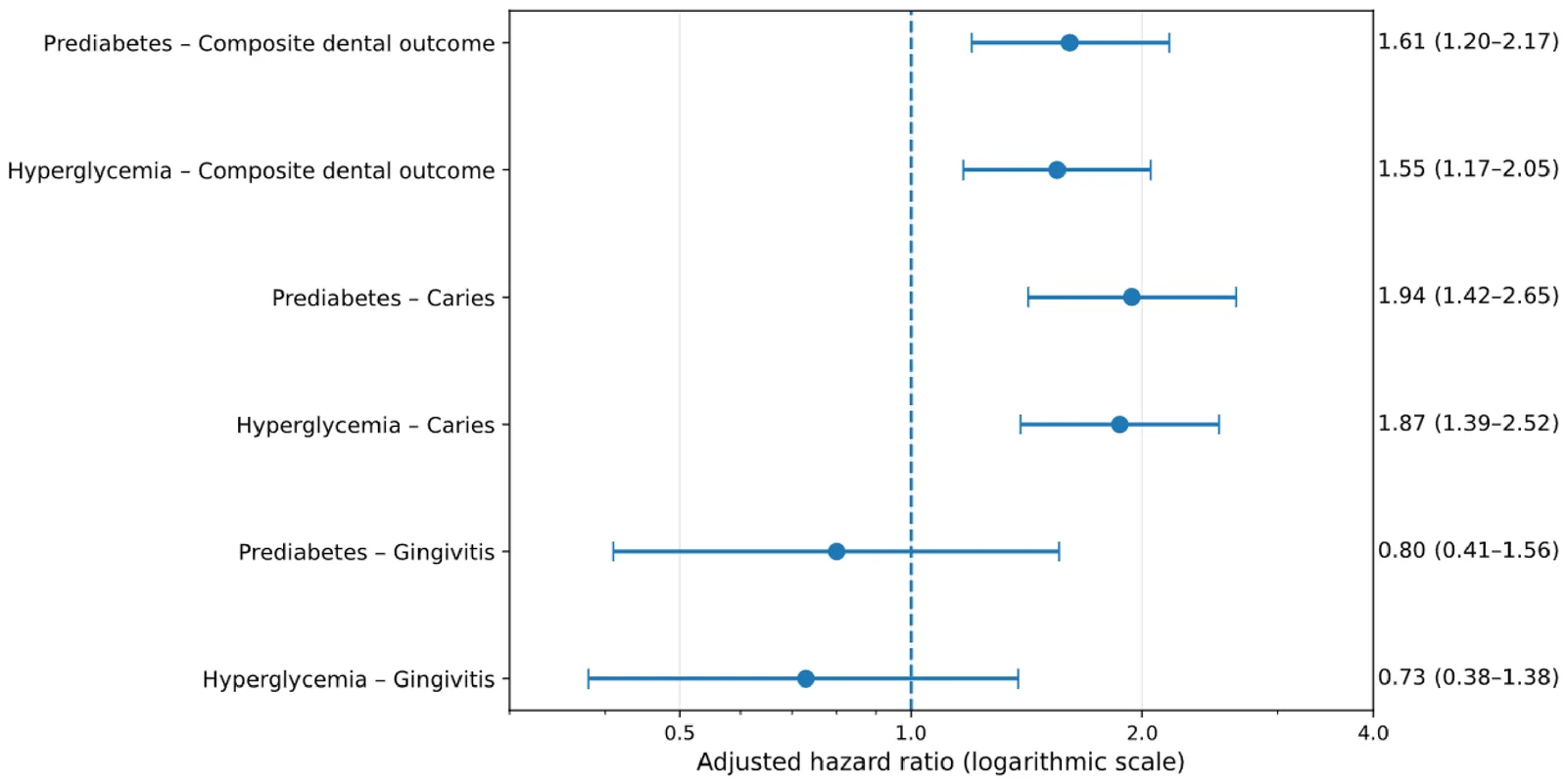
Adjusted hazard ratios for screening-based baseline prediabetes and hyperglycemia versus the composite dental outcome, incident caries, and incident gingivitis. Points represent hazard ratios adjusted for age, sex, occupation type, smoking, and alcohol consumption; horizontal bars represent 95% confidence intervals. Because proportional hazards diagnostics suggested possible time-varying associations for the composite outcome and caries, these Cox estimates should be interpreted as summary associations over follow-up.

For adjusted estimates, compared with normoglycemia, screening-based baseline prediabetes was associated with a higher hazard of the composite dental outcome in the Cox model adjusted for age, sex, occupation type, smoking, and alcohol consumption. The adjusted hazard ratio was 1.61, with a 95% CI of 1.20 to 2.17 and *p* = 0.0015. In the comparison of screening-based baseline hyperglycemia versus normoglycemia, the adjusted hazard ratio for the composite dental outcome was 1.55, with a 95% CI of 1.17 to 2.05 and *p* = 0.0021. These results are shown in Figure 2 and detailed numerically in Table S5.

Because proportional hazards diagnostics suggested possible time-varying associations for the composite outcome and incident caries, these Cox estimates should be interpreted as summary associations over follow-up rather than as constant hazard ratios throughout the entire observation period.

For secondary outcomes, the association was concentrated in incident caries. Screening-based baseline prediabetes was associated with incident caries, with an adjusted hazard ratio of 1.94, a 95% CI of 1.42 to 2.65, and *p* < 0.001. Screening-based baseline hyperglycemia was associated with incident caries, with an adjusted hazard ratio of 1.87, a 95% CI of 1.39 to 2.52, and *p* < 0.001. For incident gingivitis, adjusted estimates showed no statistically significant association. The adjusted hazard ratio was 0.80 for baseline prediabetes, with a 95% CI of 0.41 to 1.56 and *p* = 0.5155, and 0.73 for baseline hyperglycemia, with a 95% CI of 0.38 to 1.38 and *p* = 0.3254.

### 3.4. Sensitivity Analyses

In models with additional adjustment for baseline obesity, estimates were similar to those of the main model. For the composite dental outcome, the adjusted hazard ratio was 1.63 for baseline prediabetes, with a 95% CI of 1.21 to 2.19, and 1.56 for baseline hyperglycemia, with a 95% CI of 1.18 to 2.07. For incident caries, hazard ratios were 1.96 for baseline prediabetes, with a 95% CI of 1.43 to 2.68, and 1.89 for baseline hyperglycemia, with a 95% CI of 1.40 to 2.55. For incident gingivitis, estimates remained without a statistically significant association. Results of this analysis are presented in Table S6.

In the inverse-probability weighted analysis, estimates for the composite dental outcome were lower than in the conventional adjusted Cox model, although they retained a positive direction. With weighting for exposure and follow-up, the hazard ratio was 1.42 for baseline prediabetes, with a 95% CI of 1.00 to 2.00 and *p* = 0.0469, and 1.39 for baseline hyperglycemia, with a 95% CI of 1.01 to 1.91 and *p* = 0.0456. For incident caries, exposure- and follow-up-weighted hazard ratios were 1.79 for baseline prediabetes, with a 95% CI of 1.27 to 2.54, and 1.72 for baseline hyperglycemia, with a 95% CI of 1.24 to 2.39. For incident gingivitis, weighted models showed no statistically significant association. These results are presented in Table S7.

Weight diagnostics showed means close to 1 for stabilized exposure, follow-up, and combined exposure–follow-up weights. For the composite dental outcome, the 99th percentile of the combined weight was 2.43 for prediabetes and 2.73 for hyperglycemia before operational truncation. The observed maxima before truncation were 7.70 and 8.74, respectively. Additional balance diagnostics showed that weighting improved covariate balance for age, sex, occupation type, smoking, and alcohol consumption; most primary covariates had absolute standardized mean differences below 0.10 after combined IPW/IPCW weighting, although a small residual imbalance remained for sex in the prediabetes comparison. Propensity score distributions showed overlap between exposed and reference groups. The weight distribution is shown in Figure S3, and numeric summaries are presented in Table S8; balance and positivity diagnostics are presented in Tables S12 and S13.

Analytic samples varied by outcome because of outcome-specific exclusions for baseline composite dental outcome, baseline caries, or baseline gingivitis. For the composite dental outcome, models included 9804 workers and 335 events in the prediabetes comparison, and 10,050 workers and 345 events in the hyperglycemia comparison. For incident caries, models included 9839 workers and 276 events in the prediabetes comparison, and 10,086 workers and 285 events in the hyperglycemia comparison. For incident gingivitis, models included 9919 workers and 110 events in the prediabetes comparison, and 10,166 workers and 111 events in the hyperglycemia comparison. These sample sizes, events, and person-years are presented in Table S9.

Schoenfeld residual-based diagnostics suggested possible non-proportional hazards for the exposure terms in models for the composite dental outcome and incident caries, but not for the exposure terms in the gingivitis models. In time-stratified sensitivity analyses, associations with the composite dental outcome and incident caries were close to the null during the first two years and stronger beyond two years, particularly for incident caries. For incident caries, the HRs beyond two years were 2.42 for prediabetes and 2.34 for hyperglycemia, with interaction *p* values of 0.027 and 0.014, respectively. Therefore, the main Cox estimates should be interpreted as model-based summary associations over follow-up rather than as constant hazard ratios throughout the entire observation period. Results are presented in Tables S10 and S11.

## 4. Discussion

### 4.1. Main Finding

In this study of Peruvian formal-sector workers with longitudinal dental follow-up, screening-based baseline prediabetes and hyperglycemia were associated with higher recorded incidence of dental outcomes, predominantly incident caries. No increased hazard of incident gingivitis was observed. Sensitivity analyses with additional adjustment for obesity and inverse-probability weighted models retained a compatible direction, although with more attenuated estimates, suggesting that the observed caries-centered association does not depend exclusively on baseline adiposity or on the conventional Cox model. However, proportional hazards diagnostics suggested that the caries and composite estimates may vary over time; therefore, the main Cox estimates are best interpreted as summary associations over follow-up. These findings position screening-based baseline glycemic abnormalities as risk markers for recorded caries incidence in working-age adults, without assuming a direct causal interpretation.

### 4.2. Comparison with Other Studies

Our results are consistent with recent evidence linking diabetes and oral health, although they partially extend its scope. A systematic review and meta-analysis published in The Lancet Public Health described longitudinal associations between diabetes and oral diseases, with stronger evidence for periodontitis and tooth loss than for caries, and noted that oral health remains insufficiently incorporated into diabetes prevention and management strategies [16]. Unlike that study, our analysis focused on baseline prediabetes and hyperglycemia, not only established diabetes, and evaluated incident dental outcomes in a Latin American working population. Therefore, the contribution is not to confirm a classic complication of advanced diabetes, but rather to show that previous or early glycemic abnormality may also be associated with subsequent dental events.

The signal observed for incident caries also aligns with studies that have examined the cariogenic component of hyperglycemia. In the Study of Health in Pomerania, diabetes status and metabolic control were associated with longitudinal changes in coronal caries and restorative experience over 11 years of follow-up [17]. Similarly, a clinical study in adults found that blood glucose and glycated hemoglobin were associated with a greater number of root caries lesions, even after adjustment for age and salivary flow [18]. Although our study did not measure glycated hemoglobin, salivary glucose, salivary flow, or oral microbiota, the stronger association with caries rather than with gingivitis is compatible with hypotheses involving glycemic exposure, salivary composition, diet, oral pH, and cariogenic dysbiosis. These pathways should be considered plausible but not proven by our data.

The differential pattern observed for incident gingivitis should be interpreted cautiously. Lower crude rates and adjusted hazard ratios below 1 were observed among workers with prediabetes or hyperglycemia, but the confidence intervals were wide and crossed the null. Therefore, these findings should not be interpreted as evidence of a protective effect. Several explanations are plausible, including differential detection or coding at baseline, exclusion of prevalent gingivitis, low event counts among exposed workers, healthy-worker selection, unmeasured oral hygiene behavior, previous dental care, employer-level selection, and outcome misclassification. A surveillance-related explanation is possible if workers with cardiometabolic risk had more contact with occupational health services; however, surveillance intensity, prophylaxis frequency, referral, and adherence were not directly measured in this dataset. Accordingly, the gingivitis result is best interpreted as absence of increased hazard of recorded incident gingivitis and as a hypothesis-generating pattern requiring direct evaluation.

The comparison with occupational studies is particularly relevant. Evidence from a recent meta-analysis also suggests an association between prediabetes and periodontitis [19]. In Spain’s Workers’ Oral Health Study, conducted in a working population, prediabetes showed no independent association with periodontal indicators after multivariable adjustment, whereas diabetes was associated with more severe periodontitis [20]. Our study differs in three ways: it evaluated Peruvian workers, used a longitudinal design with incident outcomes, and found the main signal in caries rather than gingivitis. This difference does not invalidate either set of findings; rather, it reinforces that the relationship between glycemic abnormality and oral health depends on the dental outcome selected, the metabolic threshold used, and the measurement context. In Latin America, where nutritional transition, inequality in access to dental care, and expansion of cardiometabolic risk factors coexist, this distinction is important because associations may differ substantially according to the oral outcome, metabolic threshold, and surveillance context.

### 4.3. Public Health Implications

The public health implications should be interpreted in light of the observational design and the restricted analytic cohort. Two empirical points emerge from this study. First, among workers with longitudinal dental follow-up, screening-based baseline prediabetes and hyperglycemia were associated with higher recorded incidence of dental outcomes, mainly driven by caries. Second, only a minority of workers in the reduced occupational database had longitudinal dental outcome records, indicating limited availability of dental follow-up data and possible heterogeneity in occupational dental surveillance across employers. This pattern supports considering integrated metabolic and oral health surveillance as a candidate strategy for evaluation and strengthening systems, but it does not demonstrate that mandatory dental coverage would by itself reproduce the gingivitis pattern observed in the analytic cohort. Direct evaluation using employer-level protocol data, standardized dental outcomes, or quasi-experimental designs would be required to estimate the effect of surveillance policies on periodontal and caries outcomes.

From an international perspective, this study is relevant because global and regional oral-health strategies call for integration of oral health into noncommunicable-disease and primary health-care agendas [21,22]. It also shifts the discussion from established diabetes to early glycemic states, a stage at which prevention still has greater room to act. The International Diabetes Federation estimates that impaired fasting glucose and impaired glucose tolerance affect a substantial proportion of adults and projects increases in these conditions toward 2050, including in middle-income regions such as South and Central America [23]. If the association between prediabetes, hyperglycemia, and incident caries is confirmed in other settings, dental assessment could be incorporated more rationally into preventive packages for persons at metabolic risk. This is attractive for resource-limited health systems because it links two frequent, preventable, and measurable problems through low-complexity interventions.

The occupational setting adds another implication. Working-age adults are often left out of dental programs focused on childhood, pregnancy, or older adults, even though they have accumulated exposure to cariogenic diets, work-related stress, irregular schedules, and time barriers to accessing preventive services. Occupational evaluations do not replace primary care or clinical dentistry, but they can function as a platform for surveillance, early referral, and preventive counseling in workers with glycemic abnormalities, particularly if their effectiveness is evaluated directly. In countries with high labor informality, this approach does not cover the entire population; nevertheless, it generates useful evidence for scalable models of integration between occupational medicine, cardiometabolic prevention, and oral health.

### 4.4. Study Limitations

This study has limitations. First, only workers with longitudinal dental follow-up could contribute to the incidence analyses. Of 79,660 workers in the reduced database, 12,049 had dental follow-up records, and absence of follow-up may reflect heterogeneous implementation or recording of dental surveillance across employers. Therefore, the analytic cohort may differ from workers without dental follow-up in occupation, employer characteristics, access to dental care, health-seeking behavior, cardiometabolic risk profile, or socioeconomic position. This issue represents both an operational finding regarding limited dental outcome availability and a potential source of selection bias that limits internal validity and generalizability. Supplementary Table S14 empirically characterizes baseline differences according to longitudinal dental follow-up and baseline glycemic-classification availability, but it does not identify or quantify selection bias. Because employer-level protocol identifiers were not available in the analytic dataset, clustering by employer and direct comparison between surveillance models could not be evaluated.

Second, the study used secondary occupational records rather than standardized research examinations. Dental outcomes were clinician-recorded during routine occupational evaluations, without radiographs, examiner calibration data, periodontal probing measures, or standardized dental indices available for analysis. Misclassification of caries or gingivitis is therefore possible, especially for early lesions or mild gingival inflammation. Baseline glycemic status was based on a single fasting plasma glucose measurement, so prediabetes and hyperglycemia should be interpreted as screening-based categories rather than confirmed metabolic diagnoses. Although the exposure preceded recorded incident outcomes, reverse causality due to subclinical dental conditions not recorded at baseline, previous accumulation of undetected lesions, or shared pre-baseline determinants cannot be excluded. The database lacked information on glycated hemoglobin, duration of glycemic abnormality, treatment, salivary flow, diet, sugar intake, oral hygiene, fluoride exposure, dental visits, education, income, and precise event dates. Generalization to informal workers, unemployed persons, or the general population should be made cautiously.

## 5. Conclusions

In this retrospective occupational cohort of Peruvian formal-sector workers, screening-based baseline prediabetes and hyperglycemia were associated with higher recorded incidence of dental outcomes, mainly incident caries, among workers with longitudinal dental follow-up. No increased hazard of incident gingivitis was observed. These findings suggest that screening-based glycemic abnormalities may serve as risk markers for higher recorded caries incidence in occupational health settings and support further evaluation of integrated metabolic and oral health surveillance, but they do not establish causality or the effectiveness of dental surveillance policies.

Future studies should incorporate repeated glucose measurements, glycated hemoglobin, standardized periodontal assessments, clinical and radiographic caries evaluation, diet, oral hygiene, fluoride exposure, dental service use, employer-level protocol data, and precise event dates. Integrated metabolic and oral health surveillance in occupational settings warrants direct evaluation, particularly to determine whether it improves caries prevention, periodontal outcomes, referral, and continuity of care.

## Figures and Tables

**Table 1:** Baseline characteristics by glycemic status.

Characteristic	Total	Normoglycemia	Prediabetes	Diabetes
N	10,050	8315	1489	246
Age, mean (SD), years	37.4 (11.7)	36.2 (11.2)	42.1 (11.9)	49.8 (11.5)
Follow-up, median (IQR), years	2.1 (1.8–3.2)	2.1 (1.9–3.4)	2.1 (1.8–2.5)	2.1 (1.9–3.2)
Male sex, *n* (%)	8594 (85.5)	6996 (84.1)	1375 (92.3)	223 (90.7)
Occupation type, *n* (%)				
Office work	3894 (38.7)	3254 (39.1)	543 (36.5)	97 (39.4)
Physical/manual work	5564 (55.4)	4581 (55.1)	851 (57.2)	132 (53.7)
Customer service/sales	70 (0.7)	58 (0.7)	11 (0.7)	1 (0.4)
Health professionals	245 (2.4)	198 (2.4)	40 (2.7)	7 (2.8)
Social services	277 (2.8)	224 (2.7)	44 (3.0)	9 (3.7)
Current smoking, *n* (%)	4087 (40.7)	3287 (39.5)	678 (45.5)	122 (49.6)
Alcohol use, *n* (%)	5007 (49.8)	4187 (50.4)	702 (47.1)	118 (48.0)
BMI-defined obesity, *n* (%)	2237 (22.3)	1644 (19.8)	495 (33.2)	98 (39.8)
Baseline hypertension, *n* (%)	614 (6.1)	417 (5.0)	148 (9.9)	49 (19.9)
Composite outcome, *n* (%)	345 (3.4)	278 (3.3)	57 (3.8)	10 (4.1)
Incident caries, *n* (%)	280 (2.8)	220 (2.6)	51 (3.4)	9 (3.7)
Incident gingivitis, *n* (%)	107 (1.1)	97 (1.2)	9 (0.6)	1 (0.4)

SD: standard deviation; IQR: interquartile range. The diabetes category is presented descriptively as part of the hyperglycemia group. Composite outcome denotes incident caries or incident gingivitis.

**Table 2:** Crude incidence rates by exposure and outcome.

Outcome	Exposure Comparison	Group	N/Events/PY	Rate per 1000 PY (95% CI)
Composite outcome	Prediabetes	Normoglycemia	8315/278/22,744.6	12.22 (10.83–13.75)
Composite outcome	Prediabetes	Prediabetes	1489/57/3509.8	16.24 (12.30–21.04)
Composite outcome	Hyperglycemia	Normoglycemia	8315/278/22,744.6	12.22 (10.83–13.75)
Composite outcome	Hyperglycemia	Hyperglycemia	1735/67/4148.3	16.15 (12.52–20.51)
Incident caries	Prediabetes	Normoglycemia	8342/223/22,827.7	9.77 (8.53–11.14)
Incident caries	Prediabetes	Prediabetes	1497/53/3534.2	15.00 (11.23–19.62)
Incident caries	Hyperglycemia	Normoglycemia	8342/223/22,827.7	9.77 (8.53–11.14)
Incident caries	Hyperglycemia	Hyperglycemia	1744/62/4175.0	14.85 (11.39–19.04)
Incident gingivitis	Prediabetes	Normoglycemia	8410/100/23,013.7	4.35 (3.54–5.29)
Incident gingivitis	Prediabetes	Prediabetes	1509/10/3563.6	2.81 (1.35–5.16)
Incident gingivitis	Hyperglycemia	Normoglycemia	8410/100/23,013.7	4.35 (3.54–5.29)
Incident gingivitis	Hyperglycemia	Hyperglycemia	1756/11/4203.3	2.62 (1.31–4.68)

Rates are expressed per 1000 person-years. PY: person-years. Composite outcome denotes incident caries or incident gingivitis. Confidence intervals were calculated using an exact Poisson approximation for events observed in person-time.

## Data Availability

The anonymized database is available at https://doi.org/10.6084/m9.figshare.27098296.v1 (accessed on 6 July 2026).

## Supplementary Materials

The following supporting information can be downloaded at: https://www.mdpi.com/article/10.3390/dj14090536/s1, Table S1: STROBE checklist for cohort studies; Table S2: Operational variable dictionary; Table S3: Tabulated sample flow; Table S4: Missingness in key variables; Table S5: Numerical results underlying the Cox forest plot; Table S6: Sensitivity analysis with additional adjustment for obesity; Table S7: IPW/IPCW analysis; Table S8: Weight diagnostics; Table S9: Analytic samples by outcome; Table S10: Schoenfeld residual-based tests for the proportional hazards assumption; Table S11: Time-stratified Cox models using a 2-year cut point; Table S12: Covariate balance before and after weighting; Table S13: Positivity diagnostics using propensity-score overlap; Table S14: Baseline characteristics according to longitudinal dental follow-up availability and baseline glycemic classification availability; Figure S1: Flow diagram of the analytic sample; Figure S2: Distribution of follow-up time; Figure S3: Graphical diagnostics of stabilized IPW/IPCW weights.
