# Supplementary material for: Baseline Prediabetes, Hyperglycemia, and Incident Dental Outcomes Among Peruvian Formal-Sector Workers: A Retrospective Occupational Cohort Study
Source: Dent J (Basel). 2026 Aug 27;14(9):536. doi: 10.3390/dj14090536 (PMC13605997; doi:10.3390/dj14090536)

Supplementary Figure S2. Distribution of follow-up time.

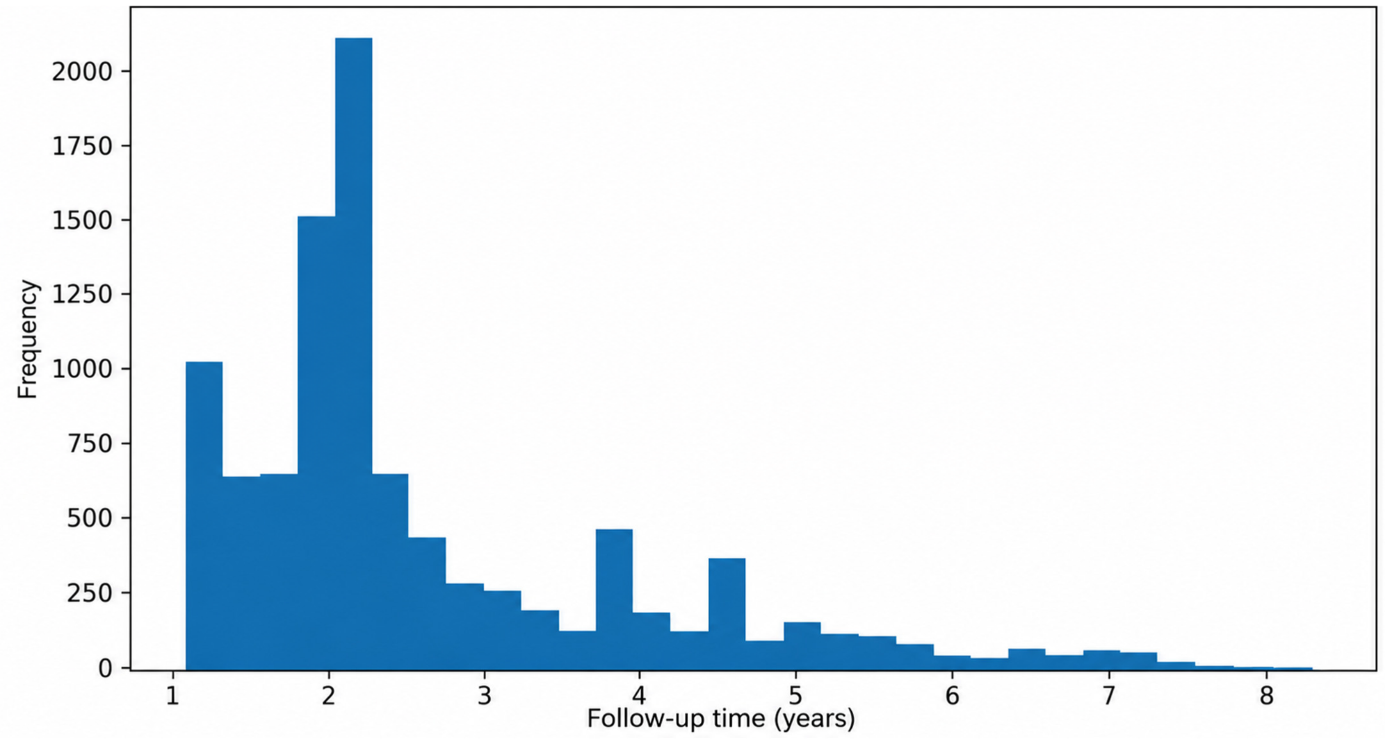

Supplement: Supplementary file 1 [file dentistry-14-00536-s001.zip › Figure S2.pdf]
