# Supplementary material for: Baseline Prediabetes, Hyperglycemia, and Incident Dental Outcomes Among Peruvian Formal-Sector Workers: A Retrospective Occupational Cohort Study
Source: Dent J (Basel). 2026 Aug 27;14(9):536. doi: 10.3390/dj14090536 (PMC13605997; doi:10.3390/dj14090536)
Supplement: Supplementary file 1 [file dentistry-14-00536-s001.zip › Table S1.pdf]

Supplementary Table S1. STROBE checklist for cohort studies.

No.	Recommendation	Location in the manuscript
**TITLE AND ABSTRACT**	**TITLE AND ABSTRACT**	**TITLE AND ABSTRACT**
1	(a) Indicate the study design with a commonly used term in the title or abstract.	Title: "Baseline Prediabetes, Hyperglycemia, and Incident Dental Outcomes among Peruvian Formal-Sector Workers: A Retrospective Occupational Cohort Study."<br><br>Abstract, Methods section: "We conducted a retrospective cohort study using occupational health records from Peruvian formal-sector workers evaluated between 2013 and 2021."<br>Single-paragraph MDPI-style abstract that integrates background, objective, methods, results, and conclusions.
	(b) Provide in the abstract an informative and balanced summary of what was done and what was found.	Reports the restricted analytic cohort (of 79,660 workers, 12,049/15.1% had longitudinal dental follow-up), the main analytic samples (n = 10,050 workers and 345 events in the hyperglycemia analysis; n = 9,804 workers and 335 events in the prediabetes analysis), and the principal findings: adjusted HR 1.61 (95% CI: 1.20-2.17) for prediabetes and the composite dental outcome, 1.55 (95% CI: 1.17-2.05) for hyperglycemia and the composite dental outcome, 1.94 (95% CI: 1.42-2.65) for prediabetes and incident caries, 1.87 (95% CI: 1.39-2.52) for hyperglycemia and incident caries, and no increased hazard of incident gingivitis.
**INTRODUCTION**	**INTRODUCTION**	**INTRODUCTION**
2	Background/rationale: explain the scientific background and rationale for the investigation being reported.	Introduction, paragraphs 1-2: global burden of oral diseases (nearly 3.7 billion people affected; untreated caries as the most frequent condition according to the Global Burden of Disease 2021); shared determinants between oral health and noncommunicable diseases; reciprocal link between diabetes and periodontal disease, with stronger

		evidence for periodontitis than for caries.<br><br>Introduction, paragraphs 3-4: regional and Peruvian context (age-standardized diabetes prevalence of 13.1% in the Americas in 2022; pooled Peruvian prevalences of diabetes of 7.47% and prediabetes of 10.66%; untreated caries in 38.2%, severe periodontitis in 19.2%, and edentulism in 14.6% of Peruvian adults) and specific evidence gap: absence of longitudinal evidence on early glycemic abnormalities and incident dental outcomes among working-age Latin American adults. The final paragraph clarifies that workers without longitudinal dental records cannot be assumed to be a directly comparable unsupervised counterfactual group.
3	Objectives: state specific objectives, including any prespecified hypotheses.	At the end of the Introduction
**METHODS**	**METHODS**	**METHODS**
4	Study design: present key elements of the design early in the paper.	Methods, subsection "Study design": observational longitudinal retrospective cohort study based on secondary records from occupational health evaluations of formal-sector workers in Peru.<br><br>Explicit adherence to STROBE recommendations for observational cohort studies (reference 10).
5	Setting: describe the setting, locations, and relevant dates, including periods of recruitment, exposure, follow-up, and data collection.	Methods, subsection "Study design": observation period corresponding to evaluations recorded between 2013 and 2021, with follow-up available until the last occupational evaluation recorded in the database.<br><br>Methods, subsection "Data source and study population": institutional database of occupational medical evaluations with sociodemographic, occupational, behavioral, anthropometric, blood pressure,

		dental evaluation, and biochemical testing information. Setting: Peru, formal-sector workers with occupational evaluation.
6	(a) Participants: state the eligibility criteria and the sources and methods of selection, as well as follow-up.	Methods, subsection "Data source and study population": workers with a baseline occupational evaluation and potential observation in a subsequent evaluation.<br><br>Inclusion: positive follow-up time, baseline dental information, records of incident dental outcomes, and available baseline glycemic classification.<br>Exclusion: caries or gingivitis recorded at baseline for the composite outcome; prevalent baseline caries for the incident caries analysis; prevalent baseline gingivitis for incident gingivitis; absence of baseline glycemic classification; baseline diabetes for the comparison of prediabetes versus normoglycemia.
	(b) Cohort: for each participant with relevant exposures and outcomes, describe exposure criteria and follow-up methods.	Not applicable
7	Variables: clearly define all outcomes, exposures, predictors, potential confounders, and effect modifiers. Give diagnostic criteria, if applicable.	Methods, subsections "Exposures," "Outcomes," and "Covariates and adjustment set."<br><br>Exposures: screening-based baseline prediabetes versus normoglycemia (excluding diabetes); baseline hyperglycemia (prediabetes or diabetes) versus normoglycemia. ADA fasting plasma glucose cutoffs: normoglycemia <100 mg/dL, prediabetes 100-125 mg/dL, diabetes >=126 mg/dL (reference 11). These are screening-based glycemic categories rather than confirmed clinical diagnoses.<br>Outcomes: composite incident dental outcome (primary; incident caries or

		incident gingivitis); incident caries and incident gingivitis (secondary). Adjustment set defined by a directed acyclic graph (Figure 1): baseline age, sex, occupation type, baseline smoking, and baseline alcohol use. Baseline obesity treated as a methodologically debatable variable and reserved for sensitivity analysis.
8*	Data sources/measurement: for each variable of interest, give the sources of data and details of assessment methods. Describe comparability of assessment methods when more than one group is involved.	Methods, subsection "Data source and study population": secondary records from occupational medical evaluations with institutionally standardized information. Methods, subsection "Exposures": fasting plasma glucose as a derived variable available in the database; absence of glycated hemoglobin, oral glucose tolerance testing, or external clinical confirmation. Methods, subsection "Outcomes": dental incidence variables recorded during the occupational evaluation; absence of an exact clinical onset date for the event. Operational definitions are detailed in Table S2.
9	Bias: describe any efforts to address potential sources of bias.	Methods, subsection "Statistical analysis": sensitivity analyses with additional adjustment for baseline obesity and inverse-probability weighted models for exposure and follow-up, with explicit assumptions of consistency, conditional exchangeability, positivity, and correct specification (references 13, 14). The proportional hazards assumption was evaluated using Schoenfeld residual-based tests, and exploratory time-stratified Cox models were fitted when diagnostics suggested possible non-proportionality.

			Discussion, limitations paragraph: explicit statement of risks of selection bias due to limited dental follow-up availability, reverse causality due to subclinical dental conditions not recorded at baseline, absence of the exact event date, reliance on routine occupational dental examinations without radiographs, periodontal probing, dental indices, or examiner calibration data, and absence of glycated hemoglobin, diet, oral hygiene, fluoride exposure, dental visits, education, and income.
10		Study size: explain how the study size was determined.	Methods, subsection "Data source and study population," and Results, subsection "Sample selection."<br><br>Study size was determined by availability: reduced database of 79,660 workers; 12,049 with positive longitudinal dental follow-up; 11,851 at risk after excluding 198 workers with caries or gingivitis at baseline; 10,050 in the hyperglycemia analysis after excluding 1,801 without glycemic classification; 9,804 in the prediabetes analysis after excluding 246 with baseline diabetes. Full flow in Figure S1 and Table S3.
11		Quantitative variables: explain how quantitative variables were handled. If applicable, describe which groupings were chosen and why.	Methods, subsection "Exposures": fasting plasma glucose categorized according to ADA cutoffs (<100, 100-125, >=126 mg/dL) to define normoglycemia, prediabetes, and diabetes. Hyperglycemia constructed as a composite category (prediabetes or diabetes) versus normoglycemia.<br><br>Rationale: alignment with international diagnostic criteria and comparability with prior literature. Age and follow-up time were retained as continuous variables in Cox models.

12	(a) Statistical methods: describe all statistical methods, including those used to control for confounding.	Methods, subsection "Statistical analysis": crude incidence rates per 1,000 person-years with 95% CIs based on the Poisson distribution; Cox proportional hazards models for hazard ratios (reference 15) adjusted for age, sex, occupation type, smoking, and alcohol use.<br><br>Adjustment set selected a priori using a directed acyclic graph (reference 12, Figure 1). Separate models for prediabetes and hyperglycemia because prediabetes is hierarchically included within hyperglycemia.
	(b) Describe any methods used to examine subgroups and interactions.	Not applicable
	(c) Explain how missing data were handled.	Methods, subsection "Statistical analysis": complete-case analysis for the indispensable variables in each comparison, without imputation, explicitly preserving the distinction between absence of a nonrequired laboratory test and absence of a variable required to define the exposure.<br><br>Results, subsection "Sample selection," and Table S4: quantitative documentation of missingness (baseline glucose with 18.0% and baseline prediabetes with 19.5% missing values in the full database).
	(d) If applicable, explain how loss to follow-up was addressed.	Methods, subsection "Statistical analysis": stabilized censoring or cohort-retention weights combined with exposure weights to obtain marginal estimates. The weighting models included age, sex, occupation type, smoking in the past 30 days, alcohol use in the past 30 days, and the corresponding glycemic exposure indicator for follow-up weights.<br><br>Results, subsection "Sensitivity analyses," and Tables S7-S8 and S12-S13: HRs with exposure and follow-up weighting; weight diagnostics with means close to 1; covariate

		balance assessed using standardized mean differences; and positivity assessed through propensity-score overlap.
		Methods, subsection "Statistical analysis": additional adjustment for baseline obesity, inverse-probability weighting for exposure and follow-up, outcome-specific dental endpoints (caries and gingivitis), proportional hazards diagnostics using Schoenfeld residuals, and exploratory time-stratified Cox models.
	(e) Describe any sensitivity analyses.	Results, subsection "Sensitivity analyses" (Tables S6-S14): HRs with obesity adjustment similar to the main model; weighted HRs retaining the same direction with attenuated magnitude; weight diagnostics in Table S8; outcome-specific sample sizes in Table S9; proportional hazards diagnostics in Table S10; time-stratified models in Table S11; covariate balance in Table S12; and positivity diagnostics in Table S13.
**RESULTS**	**RESULTS**	**RESULTS**<br>Results, subsection "Sample selection."
13*	(a) Participants: report the number of individuals at each stage of the study (potentially eligible, examined, confirmed eligible, included, with complete follow-up, and analyzed).	Reduced database: 79,660 workers -> 12,049 with positive follow-up -> 11,851 at risk (exclusion of 198 workers with caries or gingivitis at baseline) -> 10,050 in the hyperglycemia analysis (exclusion of 1,801 without glycemic classification) -> 9,804 in the prediabetes analysis (additional exclusion of 246 with baseline diabetes). The absence of longitudinal dental follow-up was described as both limited availability of dental outcome data and a potential source of selection bias. Supplementary Table S14 compares baseline characteristics according to

		availability of longitudinal dental follow-up and baseline glycemic classification.
	(b) Give reasons for nonparticipation at each stage.	Table S2
	(c) Consider use of a flow diagram.	Table S2 and Figure S1
14*	(a) Descriptive data: present characteristics of study participants (demographic, clinical, social) and information on exposures and potential confounders.	Results, subsection "Baseline characteristics," and Table 1.<br><br>Mean age 37.4 years (SD 11.7); 36.2 in normoglycemia, 42.1 in prediabetes, and 49.8 in diabetes. Male sex in 85.5%; physical work 55.4% and office work 38.7%; smoking 40.7%; alcohol use 49.8%; obesity 22.3% (19.8%, 33.2%, and 39.8% by glycemic category); hypertension 6.1% (5.0%, 9.9%, and 19.9%).
	(b) Indicate the number of participants with missing data for each variable of interest.	Table S3
	(c) Summarize follow-up time (e.g., average and total).	Results, subsection "Baseline characteristics": median follow-up of 2.1 years in the overall population and in each glycemic category (normoglycemia, prediabetes, and diabetes).<br><br>Distribution of follow-up time in Figure S2.
15*	Outcome data: report the number of outcome events or summary measures over time.	Results, subsections "Baseline characteristics" and "Crude incidence rates," and Table 2.<br><br>Events: 345 composite dental outcome events in the hyperglycemia analysis, 280 caries events, and 107 gingivitis events. Crude rates per 1,000 person-years: composite dental outcome 12.22 in normoglycemia and 16.24 in prediabetes; caries 9.77 versus 15.00; gingivitis 4.35 versus 2.81. Outcome-specific sample sizes and events in Table S9.
16	(a) Main results: give unadjusted estimates and, if applicable, confounder-adjusted estimates and	Results, subsection "Crude Incidence Rates and Adjusted Hazard Ratios," and Figure 2 (numeric values in

	their precision (95% confidence interval). Make clear which confounders were adjusted for and why they were included.	Table S5).<br><br>Composite dental outcome: adjusted HR of 1.61 (95% CI: 1.20-2.17; p = 0.0015) for prediabetes and 1.55 (95% CI: 1.17-2.05; p = 0.0021) for hyperglycemia. Incident caries: 1.94 (95% CI: 1.42-2.65; p < 0.001) for prediabetes and 1.87 (95% CI: 1.39-2.52; p < 0.001) for hyperglycemia. Incident gingivitis: 0.80 (95% CI: 0.41-1.56) and 0.73 (95% CI: 0.38-1.38), with no increased hazard observed.<br>Rationale for covariates: directed acyclic graph with epidemiologic assumptions regarding common causes of glycemic exposure and dental outcomes (Methods, subsection "Covariates and adjustment set," and Figure 1).
	(b) Report category boundaries when continuous variables were categorized.	Statistical analysis section
	(c) If relevant, consider translating estimates of relative risk into absolute risk for a meaningful time period.	Not applicable
17	Other analyses: report other analyses performed (e.g., subgroup analyses, interactions, and sensitivity analyses).	Results, subsection "Sensitivity analyses," and Tables S6-S14.<br><br>Additional adjustment for obesity: HRs of 1.63 (prediabetes) and 1.56 (hyperglycemia) for the composite dental outcome, similar to the main model.<br>Inverse-probability weighting for exposure and follow-up: attenuated HRs retaining a positive direction (1.42 for prediabetes and 1.39 for hyperglycemia for the composite dental outcome; 1.79 and 1.72 for caries). Weight diagnostics with means close to 1 and 99th percentile of 2.43-2.73 before truncation. Schoenfeld diagnostics suggested possible non-proportionality in models for the composite dental

		outcome and incident caries; time-stratified models suggested stronger associations beyond two years, particularly for incident caries.
**DISCUSSION**	**DISCUSSION**	**DISCUSSION**
18	Key results: summarize the key findings in relation to the study objectives.	Discussion section, first part
19	Limitations: discuss study limitations, considering potential sources of bias or imprecision. Discuss the direction and magnitude of any potential bias.	Discussion, limitations paragraphs: observational nature with secondary records; possible selection bias because only workers with longitudinal dental follow-up could contribute to incidence analyses; risk of reverse causality due to subclinical dental conditions not recorded at baseline; absence of an exact onset date for the dental event; outcomes derived from routine occupational evaluations without radiographs, periodontal probing, standardized dental indices, or examiner calibration data.<br><br>Unavailable variables: glycated hemoglobin, duration of glycemic abnormality, treatment, salivary flow, diet, sugar intake, oral hygiene, fluoride exposure, dental visits, education, income, employer-level protocol identifiers, and precise event dates. Population restricted to formal-sector workers with follow-up.
20	Interpretation: provide a cautious overall interpretation of results considering objectives, limitations, multiplicity of analyses, results from similar studies, and other relevant evidence.	Discussion, paragraphs 2-5: comparison with the systematic review and meta-analysis by Botelho et al. in Lancet Public Health (reference 16); Study of Health in Pomerania (reference 17); study of root caries and glycated hemoglobin (reference 18); meta-analysis of prediabetes and periodontitis (reference 19); Workers' Oral Health Study in Spain (reference 20).

		Cautious interpretation: estimates are interpreted as associations between screening-based baseline glycemic abnormality and incident detection of dental outcomes during occupational follow-up, without definitive causal inference. The gingivitis finding is interpreted as absence of increased hazard of recorded incident gingivitis and as hypothesis-generating rather than as evidence of protection or operational effectiveness.
		Discussion, limitations paragraph: the population was restricted to formal-sector workers with follow-up; therefore, generalization to informal workers, unemployed persons, or the general population should be made cautiously.
21	Generalizability: discuss the generalizability (external validity) of the study results.	Discussion, paragraphs "Public health implications" and "international perspective": applicability to middle-income health systems undergoing epidemiologic transition and expansion of cardiometabolic risk factors; alignment with the WHO global strategy and action plan on oral health 2023-2030 (references 21, 22, 23).
**OTHER INFORMATION**	**OTHER INFORMATION**	**OTHER INFORMATION**
22	Funding: indicate the source of funding and the role of funders in the current study and, if applicable, in the original study on which the present article is based.	The study was self-funded

STROBE = Strengthening the Reporting of Observational Studies in Epidemiology.
