# Supplementary material for: Baseline Prediabetes, Hyperglycemia, and Incident Dental Outcomes Among Peruvian Formal-Sector Workers: A Retrospective Occupational Cohort Study
Source: Dent J (Basel). 2026 Aug 27;14(9):536. doi: 10.3390/dj14090536 (PMC13605997; doi:10.3390/dj14090536)
Supplement: Supplementary file 1 [file dentistry-14-00536-s001.zip › Table S10.pdf]

Supplementary Table S10. Schoenfeld residual-based tests for the proportional hazards assumption.

Outcome	Exposure	N	Events	HR (95% CI)	Exposure PH p	Global PH p	Conclusion
Composite dental outcome	Baseline prediabetes	9,804	335	1.61 (1.20-2.17)	0.008	<0.001	Potential violation for exposure term
Composite dental outcome	Baseline hyperglycemia	10,050	345	1.55 (1.17-2.05)	0.001	<0.001	Potential violation for exposure term
Incident caries	Baseline prediabetes	9,839	276	1.94 (1.42-2.65)	0.005	<0.001	Potential violation for exposure term
Incident caries	Baseline hyperglycemia	10,086	285	1.87 (1.39-2.52)	<0.001	<0.001	Potential violation for exposure term
Incident gingivitis	Baseline prediabetes	9,919	110	0.80 (0.41-1.56)	0.685	0.037	Exposure term acceptable; possible global non-proportionality
Incident gingivitis	Baseline hyperglycemia	10,166	111	0.73 (0.38-1.38)	0.880	0.051	No evidence of material violation

PH: proportional hazards. Models were adjusted for age, sex, occupation type, smoking, and alcohol consumption.
