# Supplementary material for: Baseline Prediabetes, Hyperglycemia, and Incident Dental Outcomes Among Peruvian Formal-Sector Workers: A Retrospective Occupational Cohort Study
Source: Dent J (Basel). 2026 Aug 27;14(9):536. doi: 10.3390/dj14090536 (PMC13605997; doi:10.3390/dj14090536)
Supplement: Supplementary file 1 [file dentistry-14-00536-s001.zip › Table S11.pdf]

Supplementary Table S11. Time-stratified Cox models using a 2-year cut point.

Outcome	Exposure	N persons	Events total	Events <=2 / >2 years	HR <=2 years (95% CI)	HR >2 years (95% CI)	Interaction p
Composite dental outcome	Baseline prediabetes	9,804	335	77 / 258	1.05 (0.58-1.92)	1.88 (1.35-2.63)	0.095
Composite dental outcome	Baseline hyperglycemia	10,050	345	79 / 266	1.02 (0.58-1.80)	1.80 (1.31-2.47)	0.083
Incident caries	Baseline prediabetes	9,839	276	66 / 210	1.06 (0.55-2.03)	2.42 (1.70-3.43)	0.027
Incident caries	Baseline hyperglycemia	10,086	285	67 / 218	0.97 (0.52-1.82)	2.34 (1.68-3.26)	0.014
Incident gingivitis	Baseline prediabetes	9,919	110	22 / 88	0.86 (0.25-2.91)	0.78 (0.36-1.71)	0.902
Incident gingivitis	Baseline hyperglycemia	10,166	111	23 / 88	0.97 (0.33-2.88)	0.64 (0.29-1.40)	0.533

The 2-year cut point was selected for interpretability and because it approximated the median follow-up. Models were adjusted for age, sex, occupation type, smoking, and alcohol consumption.
