# Supplementary material for: Baseline Prediabetes, Hyperglycemia, and Incident Dental Outcomes Among Peruvian Formal-Sector Workers: A Retrospective Occupational Cohort Study
Source: Dent J (Basel). 2026 Aug 27;14(9):536. doi: 10.3390/dj14090536 (PMC13605997; doi:10.3390/dj14090536)
Supplement: Supplementary file 1 [file dentistry-14-00536-s001.zip › Table S12.pdf]

Supplementary Table S12. Covariate balance before and after weighting.

Exposure comparison	Covariate	Unweighted SMD	After exposure IPW	After combined IPW/IPCW	Interpretation
Prediabetes vs normoglycemia	Age, years	0.515	0.054	0.088	Balanced (<0.10)
Prediabetes vs normoglycemia	Sex (=1)	0.257	0.069	0.114	Residual imbalance
Prediabetes vs normoglycemia	Occupation type (=1)	0.045	0.008	0.007	Balanced (<0.10)
Prediabetes vs normoglycemia	Smoking in past 30 days (=1)	0.122	0.019	0.035	Balanced (<0.10)
Prediabetes vs normoglycemia	Alcohol use in past 30 days (=1)	-0.064	-0.019	-0.038	Balanced (<0.10)
Prediabetes vs normoglycemia	Baseline obesity (sensitivity variable)	0.309	0.254	0.264	Not balanced; sensitivity covariate not included in primary weights
Hyperglycemia vs normoglycemia	Age, years	0.603	0.042	0.068	Balanced (<0.10)
Hyperglycemia vs normoglycemia	Sex (=1)	0.248	0.055	0.094	Balanced (<0.10)
Hyperglycemia vs normoglycemia	Occupation type (=1)	0.035	0.000	-0.002	Balanced (<0.10)
Hyperglycemia vs normoglycemia	Smoking in past 30 days (=1)	0.133	0.021	0.039	Balanced (<0.10)
Hyperglycemia vs normoglycemia	Alcohol use in past 30 days (=1)	-0.062	-0.017	-0.033	Balanced (<0.10)
Hyperglycemia vs normoglycemia	Baseline obesity (sensitivity variable)	0.329	0.264	0.276	Not balanced; sensitivity covariate not included in primary weights

SMD: standardized mean difference. IPW: inverse probability weighting. IPCW: inverse probability of censoring/follow-up weighting.
