# Supplementary material for: Baseline Prediabetes, Hyperglycemia, and Incident Dental Outcomes Among Peruvian Formal-Sector Workers: A Retrospective Occupational Cohort Study
Source: Dent J (Basel). 2026 Aug 27;14(9):536. doi: 10.3390/dj14090536 (PMC13605997; doi:10.3390/dj14090536)
Supplement: Supplementary file 1 [file dentistry-14-00536-s001.zip › Table S13.pdf]

Supplementary Table S13. Positivity diagnostics using propensity-score overlap.

Exposure comparison	Group	N	Propensity score min	p1	p50	p99	max
Prediabetes vs normoglycemia	Reference	8,315	0.032	0.041	0.128	0.360	0.468
Prediabetes vs normoglycemia	Exposed	1,489	0.035	0.052	0.175	0.384	0.475
Hyperglycemia vs normoglycemia	Reference	8,315	0.032	0.043	0.137	0.427	0.560
Hyperglycemia vs normoglycemia	Exposed	1,735	0.036	0.058	0.206	0.473	0.597

Propensity-score distributions showed overlap between exposed and reference groups, without evidence of complete separation. Stabilized weight diagnostics are reported in Supplementary Table S8.
