# Supplementary material for: Baseline Prediabetes, Hyperglycemia, and Incident Dental Outcomes Among Peruvian Formal-Sector Workers: A Retrospective Occupational Cohort Study
Source: Dent J (Basel). 2026 Aug 27;14(9):536. doi: 10.3390/dj14090536 (PMC13605997; doi:10.3390/dj14090536)
Supplement: Supplementary file 1 [file dentistry-14-00536-s001.zip › Table S14.pdf]

Supplementary Table S14. Baseline characteristics according to longitudinal dental follow-up availability and baseline glycemic classification availability.

Panel A. Workers with versus without longitudinal dental follow-up.

Characteristic	With follow-up N = 12,049	Without follow-up N = 67,611	Absolute SMD
Age, years, mean (SD)	37.2 (11.6)	32.9 (10.2)	0.390
Male sex, n (%)	10,226 (84.9)	52,860 (78.2)	0.173
**Occupation type, n (%)**			
Office work	4,357 (36.2)	24,229 (35.8)	0.007
Physical/manual work	6,584 (54.6)	37,668 (55.7)	0.022
Customer service/sales	80 (0.7)	1,436 (2.1)	0.125
Health professionals	284 (2.4)	2,662 (3.9)	0.091
Social services	744 (6.2)	1,609 (2.4)	0.188
Missing	0 (0.0)	7 (0.0)	0.014
Current smoking in previous 30 days, n (%)	4,935 (41.0)	25,617 (37.9)	0.063
Alcohol use in previous 30 days, n (%)	6,018 (49.9)	34,634 (51.2)	0.026
Night work, n (%)	794 (6.6)	3,075 (4.5)	0.089
Prolonged sitting/seated work, n (%)	5,849 (48.5)	26,088 (38.6)	0.202
Family history of diabetes, n (%)	1,260 (10.5)	9,185 (13.6)	0.096
Family history of hypertension, n (%)	3,189 (26.5)	18,276 (27.0)	0.013
Baseline caries, n (%)	161 (1.3)	1,616 (2.4)	0.078
Baseline gingivitis, n (%)	65 (0.5)	555 (0.8)	0.034
Baseline caries or gingivitis, n (%)	198 (1.6)	1,837 (2.7)	0.074
Body mass index, kg/m2, mean (SD)	27.2 (4.0)	26.8 (4.2)	0.079
Baseline obesity by BMI, n (%)	2,601 (21.6)	14,093 (20.8)	0.018
Waist circumference available, n (%)	10,300 (85.5)	57,258 (84.7)	0.022
Waist circumference, cm, mean (SD)	91.1 (10.4)	89.1 (10.7)	0.194
Systolic blood pressure, mmHg, mean (SD)	111.3 (12.3)	108.7 (10.4)	0.222
Diastolic blood pressure, mmHg, mean (SD)	71.9 (13.3)	70.6 (7.8)	0.124
Baseline hypertension, n (%)	698 (5.8)	572 (0.8)	0.279
Baseline glucose available, n (%)	10,230 (84.9)	55,060 (81.4)	0.093
Fasting glucose, mg/dL, median (IQR)	91.0 (83.0-98.0)	90.0 (82.0-97.0)	0.065
**Baseline glycemic status among workers with glucose, n (%)**			
Normoglycemia	8,461 (82.7)	46,328 (84.1)	0.039
Prediabetes	1,521 (14.9)	7,799 (14.2)	0.020
Diabetes	248 (2.4)	933 (1.7)	0.051
Baseline cholesterol available, n (%)	5,914 (49.1)	30,137 (44.6)	0.090
Total cholesterol, mg/dL, median (IQR)	191.0 (167.0-220.0)	182.0 (159.0-206.0)	0.246
Baseline hypercholesterolemia, n (%)	2,474 (41.8)	9,624 (31.9)	0.206
Baseline triglycerides available, n (%)	5,946 (49.3)	29,353 (43.4)	0.119
Triglycerides, mg/dL, median (IQR)	124.0 (89.0-178.0)	114.0 (85.0-159.0)	0.150
Baseline hypertriglyceridemia, n (%)	2,166 (36.4)	8,768 (29.9)	0.140
Baseline dyslipidemia, n (%)	1,414 (11.7)	5,056 (7.5)	0.145
Baseline stress, n (%)	181 (1.5)	918 (1.4)	0.012
Baseline depression, n (%)	106 (0.9)	755 (1.1)	0.024
Baseline anxiety, n (%)	39 (0.3)	369 (0.5)	0.034

Note. Values are mean (SD), median (IQR), or n (%), as indicated. Standardized mean differences are absolute values. Percentages are calculated among workers with non-missing values unless otherwise specified; for availability indicators, percentages use the full group denominator. For continuous variables summarized as median (IQR), standardized mean differences were calculated on the original continuous scale. For multicategory variables, standardized mean differences are presented for each category versus all other categories. No p-values were calculated.

Supplementary Table S14. Baseline characteristics according to longitudinal dental follow-up availability and baseline glycemic classification availability.

Panel B. Workers with versus without baseline glycemic classification among those with follow-up and no baseline caries or gingivitis.

Characteristic	With glycemic classification N = 10,050	Without glycemic classification N = 1,801	Absolute SMD
Age, years, mean (SD)	37.4 (11.7)	36.2 (10.9)	0.108
Male sex, n (%)	8,594 (85.5)	1,446 (80.3)	0.139
**Occupation type, n (%)**			
Office work	3,894 (38.7)	400 (22.2)	0.365
Physical/manual work	5,564 (55.4)	896 (49.8)	0.113
Customer service/sales	70 (0.7)	10 (0.6)	0.018
Health professionals	245 (2.4)	34 (1.9)	0.038
Social services	277 (2.8)	461 (25.6)	0.693
Current smoking in previous 30 days, n (%)	4,087 (40.7)	766 (42.5)	0.038
Alcohol use in previous 30 days, n (%)	5,007 (49.8)	912 (50.6)	0.016
Night work, n (%)	665 (6.6)	68 (3.8)	0.128
Prolonged sitting/seated work, n (%)	5,015 (49.9)	728 (40.4)	0.191
Family history of diabetes, n (%)	1,048 (10.4)	190 (10.5)	0.004
Family history of hypertension, n (%)	2,739 (27.3)	411 (22.8)	0.102
Body mass index, kg/m2, mean (SD)	27.2 (4.0)	26.6 (3.9)	0.153
Baseline obesity by BMI, n (%)	2,237 (22.3)	323 (17.9)	0.108
Waist circumference available, n (%)	8,450 (84.1)	1,701 (94.4)	0.340
Waist circumference, cm, mean (SD)	91.5 (10.5)	89.4 (10.0)	0.204
Systolic blood pressure, mmHg, mean (SD)	111.6 (12.3)	109.3 (12.0)	0.189
Diastolic blood pressure, mmHg, mean (SD)	72.3 (14.0)	70.0 (8.8)	0.191
Baseline hypertension, n (%)	614 (6.1)	74 (4.1)	0.091
Baseline cholesterol available, n (%)	5,126 (51.0)	704 (39.1)	0.241
Total cholesterol, mg/dL, median (IQR)	190.0 (166.0-218.0)	197.0 (174.0-220.0)	0.141
Baseline hypercholesterolemia, n (%)	2,104 (41.0)	332 (47.2)	0.123
Baseline triglycerides available, n (%)	5,138 (51.1)	700 (38.9)	0.248
Triglycerides, mg/dL, median (IQR)	124.0 (89.0-177.0)	126.0 (90.0-180.0)	0.043
Baseline hypertriglyceridemia, n (%)	1,863 (36.3)	256 (36.6)	0.006
Baseline dyslipidemia, n (%)	1,211 (12.0)	179 (9.9)	0.068
Baseline stress, n (%)	167 (1.7)	9 (0.5)	0.113
Baseline depression, n (%)	95 (0.9)	8 (0.4)	0.060
Baseline anxiety, n (%)	37 (0.4)	2 (0.1)	0.053
**Follow-up descriptor**			
Follow-up time, years, median (IQR)	2.1 (1.8-3.2)	2.5 (1.9-3.9)	0.110

Note. Values are mean (SD), median (IQR), or n (%), as indicated. Standardized mean differences are absolute values. Percentages are calculated among workers with non-missing values unless otherwise specified; for availability indicators, percentages use the full group denominator. For continuous variables summarized as median (IQR), standardized mean differences were calculated on the original continuous scale. For multicategory variables, standardized mean differences are presented for each category versus all other categories. No p-values were calculated. Panel B is restricted to workers with longitudinal dental follow-up and no baseline caries or gingivitis; therefore, baseline dental outcome variables are not displayed because they are constant by design. Follow-up time is presented only as a descriptive follow-up characteristic.

Interpretive boundary. These tables describe differential availability of follow-up and baseline glycemic classification. They should be interpreted as an empirical characterization of selection into the analytic sample, not as a correction for employer-level surveillance heterogeneity or unmeasured determinants of follow-up, which remain limitations in the absence of employer identifiers and detailed protocol information.
