# Supplementary material for: Baseline Prediabetes, Hyperglycemia, and Incident Dental Outcomes Among Peruvian Formal-Sector Workers: A Retrospective Occupational Cohort Study
Source: Dent J (Basel). 2026 Aug 27;14(9):536. doi: 10.3390/dj14090536 (PMC13605997; doi:10.3390/dj14090536)
Supplement: Supplementary file 1 [file dentistry-14-00536-s001.zip › Table S2.pdf]

Supplementary Table S2. Operational variable dictionary.

Variable	Role	Operational definition
prediabetes_base / prediabetes	Main exposure	Baseline prediabetes, compared with normoglycemia; baseline diabetes excluded from this comparison.
hiperglicemia_base / hyperglycemia_any	Secondary main exposure	Baseline hyperglycemia: baseline prediabetes or diabetes; reference: normoglycemia.
gly_status	Descriptive variable	0=normoglycemia; 1=prediabetes; 2=diabetes within the hyperglycemic group.
enfermedad_dental_incidente	Primary outcome	Incident caries or incident gingivitis during follow-up; baseline caries or gingivitis is excluded.
caries_incidente	Secondary outcome	Incident caries during follow-up; prevalent baseline caries is excluded.
gingivitis_incidente	Secondary outcome	Incident gingivitis during follow-up; prevalent baseline gingivitis is excluded.
tiempo_seguimiento / time_years	Analysis time	Follow-up time divided by 12 to express person-years.
edad1	DAG confounder	Baseline age in years.
sexo	DAG confounder	Baseline sex, coded according to the source database.
tipo_de_ocupacion_2	DAG confounder	Binary occupation type; used in adjusted models because of separation in detailed occupational categories.
fumador1	DAG confounder	Baseline smoking coded 1 vs 0.
consumo_alcohol1	DAG confounder	Baseline alcohol use coded 1 vs 0.
obesidad_base	Sensitivity	Baseline BMI-defined obesity; included only in sensitivity analyses because of possible confounding/common metabolism.

Operational definitions correspond to the longitudinal analytic database used for this manuscript.
