# Supplementary material for: Baseline Prediabetes, Hyperglycemia, and Incident Dental Outcomes Among Peruvian Formal-Sector Workers: A Retrospective Occupational Cohort Study
Source: Dent J (Basel). 2026 Aug 27;14(9):536. doi: 10.3390/dj14090536 (PMC13605997; doi:10.3390/dj14090536)
Supplement: Supplementary file 1 [file dentistry-14-00536-s001.zip › Table S3.pdf]

Supplementary Table S3. Tabulated sample flow.

Step	N	Excluded	Reason
Reduced database available	79,660	—	Workers in the reduced database
With positive longitudinal follow-up	12,049	67,611	No follow-up or unavailable follow-up time
Free of baseline caries or gingivitis	11,851	198	Prevalent baseline caries or gingivitis
With baseline glycemic classification	10,050	1,801	Baseline glucose/glycemic classification unavailable
Prediabetes vs normoglycemia analysis	9,804	246	Baseline diabetes excluded from the prediabetes comparison
Hyperglycemia vs normoglycemia analysis	10,050	0	Hyperglycemia includes prediabetes and diabetes

The sample flow corresponds to the main analysis of baseline prediabetes/hyperglycemia and composite dental outcome.
