# Supplementary material for: Baseline Prediabetes, Hyperglycemia, and Incident Dental Outcomes Among Peruvian Formal-Sector Workers: A Retrospective Occupational Cohort Study
Source: Dent J (Basel). 2026 Aug 27;14(9):536. doi: 10.3390/dj14090536 (PMC13605997; doi:10.3390/dj14090536)
Supplement: Supplementary file 1 [file dentistry-14-00536-s001.zip › Table S4.pdf]

Supplementary Table S4. Missingness in key variables.

Variable	Description	Total N	Missing	% missing	Nonmissing
tiene_seguimiento	Longitudinal follow-up available	79,660	0	0	79,660
tiempo_seguimiento	Follow-up time	79,660	67,611	84.87	12,049
enfermedad_dental_base	Baseline caries or gingivitis	79,660	0	0	79,660
caries_base	Prevalent baseline caries	79,660	0	0	79,660
gingivitis_base	Prevalent baseline gingivitis	79,660	0	0	79,660
enfermedad_dental_incidente	Composite composite dental outcome	79,660	67,611	84.87	12,049
caries_incidente	Incident caries	79,660	67,611	84.87	12,049
gingivitis_incidente	Incident gingivitis	79,660	67,611	84.87	12,049
glucosa_base	Baseline glucose	79,660	14,370	18.04	65,290
prediabetes_base	Prediabetes	79,660	15,551	19.52	64,109
hiperglicemia_base	Hyperglycemia	79,660	14,370	18.04	65,290
diabetes_base	Baseline diabetes	79,660	14,370	18.04	65,290
edad1	Age	79,660	0	0	79,660
sexo	Sex	79,660	0	0	79,660
tipo_de_ocupacion	Occupation type	79,660	7	0.01	79,653
tipo_de_ocupacion_2	Binary occupation type	79,660	7	0.01	79,653
fumador1	Baseline smoking	79,660	0	0	79,660
consumo_alcohol1	Baseline alcohol use	79,660	0	0	79,660
obesidad_base	Baseline obesity	79,660	1	0.00	79,659
hta_base	Baseline hypertension	79,660	0	0	79,660

Missingness was calculated on the reduced available database of 79,660 workers.
