# Supplementary material for: Baseline Prediabetes, Hyperglycemia, and Incident Dental Outcomes Among Peruvian Formal-Sector Workers: A Retrospective Occupational Cohort Study
Source: Dent J (Basel). 2026 Aug 27;14(9):536. doi: 10.3390/dj14090536 (PMC13605997; doi:10.3390/dj14090536)
Supplement: Supplementary file 1 [file dentistry-14-00536-s001.zip › Table S5.pdf]

Supplementary Table S5. Numerical results underlying the Cox forest plot.

Outcome	Exposure	N	Events	HR (95% CI)	p	Model
Composite outcome	Prediabetes	9,804	335	1.61 (1.20-2.17)	0.002	DAG-adjusted Cox
Composite outcome	Hyperglycemia	10,050	345	1.55 (1.17-2.05)	0.002	DAG-adjusted Cox
Incident caries	Prediabetes	9,839	276	1.94 (1.42-2.65)	0	DAG-adjusted Cox
Incident caries	Hyperglycemia	10,086	285	1.87 (1.39-2.52)	0	DAG-adjusted Cox
Incident gingivitis	Prediabetes	9,919	110	0.80 (0.41-1.56)	0.515	DAG-adjusted Cox
Incident gingivitis	Hyperglycemia	10,166	111	0.73 (0.38-1.38)	0.325	DAG-adjusted Cox

Cox models were adjusted for age, sex, occupation type, smoking, and alcohol use. Reference group: normoglycemia. Composite outcome denotes incident caries or incident gingivitis.
