# Supplementary material for: Baseline Prediabetes, Hyperglycemia, and Incident Dental Outcomes Among Peruvian Formal-Sector Workers: A Retrospective Occupational Cohort Study
Source: Dent J (Basel). 2026 Aug 27;14(9):536. doi: 10.3390/dj14090536 (PMC13605997; doi:10.3390/dj14090536)
Supplement: Supplementary file 1 [file dentistry-14-00536-s001.zip › Table S6.pdf]

Supplementary Table S6. Sensitivity analysis with additional adjustment for obesity.

Outcome	Exposure	N	Events	HR (95% CI)	p	Model
Composite outcome	Prediabetes	9,804	335	1.63 (1.21-2.19)	0.001	Cox + obesity
Composite outcome	Hyperglycemia	10,050	345	1.56 (1.18-2.07)	0.002	Cox + obesity
Incident caries	Prediabetes	9,839	276	1.96 (1.43-2.68)	0	Cox + obesity
Incident caries	Hyperglycemia	10,086	285	1.89 (1.40-2.55)	0	Cox + obesity
Incident gingivitis	Prediabetes	9,919	110	0.80 (0.41-1.55)	0.504	Cox + obesity
Incident gingivitis	Hyperglycemia	10,166	111	0.72 (0.38-1.36)	0.310	Cox + obesity

Cox models were adjusted for the minimum adjustment set plus baseline obesity. Reference group: normoglycemia. Composite outcome denotes incident caries or incident gingivitis.
