# Supplementary material for: Baseline Prediabetes, Hyperglycemia, and Incident Dental Outcomes Among Peruvian Formal-Sector Workers: A Retrospective Occupational Cohort Study
Source: Dent J (Basel). 2026 Aug 27;14(9):536. doi: 10.3390/dj14090536 (PMC13605997; doi:10.3390/dj14090536)
Supplement: Supplementary file 1 [file dentistry-14-00536-s001.zip › Table S7.pdf]

Supplementary Table S7. IPW/IPCW analysis.

Outcome	Exposure	Weighting model	N	Events	HR (95% CI)	p
Composite outcome	Prediabetes	Exposure IPW	9,804	335	1.52 (1.09-2.12)	0.014
Composite outcome	Prediabetes	Exposure + follow-up IPW	9,804	335	1.42 (1.00-2.00)	0.047
Composite outcome	Hyperglycemia	Exposure IPW	10,050	345	1.48 (1.08-2.03)	0.014
Composite outcome	Hyperglycemia	Exposure + follow-up IPW	10,050	345	1.39 (1.01-1.91)	0.046
Incident caries	Prediabetes	Exposure IPW	9,839	276	1.86 (1.32-2.63)	<0.001
Incident caries	Prediabetes	Exposure + follow-up IPW	9,839	276	1.79 (1.27-2.54)	<0.001
Incident caries	Hyperglycemia	Exposure IPW	10,086	285	1.80 (1.30-2.49)	<0.001
Incident caries	Hyperglycemia	Exposure + follow-up IPW	10,086	285	1.72 (1.24-2.39)	0.001
Incident gingivitis	Prediabetes	Exposure IPW	9,919	110	0.80 (0.38-1.68)	0.559
Incident gingivitis	Prediabetes	Exposure + follow-up IPW	9,919	110	0.79 (0.38-1.67)	0.543
Incident gingivitis	Hyperglycemia	Exposure IPW	10,166	111	0.78 (0.39-1.56)	0.482
Incident gingivitis	Hyperglycemia	Exposure + follow-up IPW	10,166	111	0.80 (0.41-1.58)	0.525

IPW: inverse probability weighting; IPCW: inverse probability of censoring/follow-up weighting. Reference group: normoglycemia. Composite outcome denotes incident caries or incident gingivitis.
