# Supplementary material for: Baseline Prediabetes, Hyperglycemia, and Incident Dental Outcomes Among Peruvian Formal-Sector Workers: A Retrospective Occupational Cohort Study
Source: Dent J (Basel). 2026 Aug 27;14(9):536. doi: 10.3390/dj14090536 (PMC13605997; doi:10.3390/dj14090536)
Supplement: Supplementary file 1 [file dentistry-14-00536-s001.zip › Table S8.pdf]

Supplementary Table S8. Weight diagnostics.

Outcome	Exposure	Weight	mean	sd	min	p1	p50	p99	max	tr_min	tr_max
Composite outcome	Prediabetes	sw_E	1.00	0.19	0.30	0.42	0.99	1.54	3.18	0.42	1.54
Composite outcome	Prediabetes	sw_R	1.00	0.32	0.37	0.46	0.98	1.83	2.42	0.46	1.83
Composite outcome	Prediabetes	sw_ER	1.00	0.39	0.12	0.24	0.96	2.43	7.70	0.24	2.43
Composite outcome	Hyperglycemia	sw_E	1.00	0.23	0.27	0.38	0.98	1.70	3.51	0.38	1.70
Composite outcome	Hyperglycemia	sw_R	1.00	0.32	0.37	0.46	0.97	1.83	2.49	0.46	1.83
Composite outcome	Hyperglycemia	sw_ER	1.00	0.43	0.11	0.21	0.95	2.73	8.73	0.21	2.73
Incident caries	Prediabetes	sw_E	1.00	0.19	0.30	0.42	0.99	1.54	3.18	0.42	1.54
Incident caries	Prediabetes	sw_R	1.00	0.32	0.37	0.46	0.98	1.83	2.42	0.46	1.83
Incident caries	Prediabetes	sw_ER	1.00	0.39	0.12	0.24	0.96	2.44	7.70	0.24	2.44
Incident caries	Hyperglycemia	sw_E	1.00	0.23	0.27	0.38	0.98	1.70	3.51	0.38	1.70
Incident caries	Hyperglycemia	sw_R	1.00	0.32	0.37	0.46	0.97	1.83	2.49	0.46	1.83
Incident caries	Hyperglycemia	sw_ER	1.00	0.43	0.11	0.21	0.95	2.76	8.73	0.21	2.76
Incident gingivitis	Prediabetes	sw_E	1.00	0.19	0.30	0.43	0.99	1.53	3.14	0.43	1.53
	Prediabetes	sw_R	1.00	0.32	0.37	0.46	0.98	1.83	2.42	0.46	1.83
	Prediabetes	sw_ER	1.00	0.39	0.12	0.24	0.96	2.40	7.61	0.24	2.40
	Hyperglycemia	sw_E	1.00	0.23	0.27	0.38	0.98	1.69	3.47	0.38	1.69
	Hyperglycemia	sw_R	1.00	0.32	0.37	0.46	0.97	1.83	2.49	0.46	1.83
	Hyperglycemia	sw_ER	1.00	0.43	0.11	0.21	0.95	2.72	8.63	0.21	2.72

sw_E: stabilized exposure weight; sw_R: stabilized follow-up weight; sw_ER: combined exposure-follow-up weight.

Composite outcome denotes incident caries or incident gingivitis.
