# Supplementary material for: Baseline Prediabetes, Hyperglycemia, and Incident Dental Outcomes Among Peruvian Formal-Sector Workers: A Retrospective Occupational Cohort Study
Source: Dent J (Basel). 2026 Aug 27;14(9):536. doi: 10.3390/dj14090536 (PMC13605997; doi:10.3390/dj14090536)
Supplement: Supplementary file 1 [file dentistry-14-00536-s001.zip › Table S9.pdf]

Supplementary Table S9. Analytic samples by outcome.

Outcome	Exposure	Analytic N	Events	Person-years
Composite outcome	Prediabetes	9,804	335	26,254.4
Composite outcome	Hyperglycemia	10,050	345	26,892.9
Incident caries	Prediabetes	9,839	276	26,361.9
Incident caries	Hyperglycemia	10,086	285	27,002.7
Incident gingivitis	Prediabetes	9,919	110	26,577.3
Incident gingivitis	Hyperglycemia	10,166	111	27,217

Samples vary according to exclusions specific to the corresponding baseline outcome. Composite outcome denotes incident caries or incident gingivitis.
